# Ionic Liquids under Radiation and the Dimer Radical Dicyanamide Anion

**DOI:** 10.1021/acs.jpcb.5c07381

**Published:** 2025-11-13

**Authors:** Hung H. Nguyen, Katie Huber, Dishan Das, James F. Wishart, David A. Blank, Claudio J. Margulis

**Affiliations:** † Department of Chemistry, 4083 The University of Iowa, Iowa City, Iowa 52242, United States; ‡ Department of Chemistry, 5635 University of Minnesota, Minneapolis, Minnesota 55455, United States; ¶ Chemistry Department, 8099 Brookhaven National Laboratory, Upton, New York 11973, United States

## Abstract

Ionic liquids (ILs) used in solar, electrochemical, nuclear, or even space exploration applications will necessarily be exposed to conditions in which electron detachment may occur and transient charge-depleted radical species may form. In a recent article (*J. Am. Chem. Soc.* **2025**, 147, 23395–23398), we studied the different possible kinetic fates of an excess electron in the popular 1-butyl-1-methylpyrrolidinium dicyanamide IL but did not address the nature of electron-deficient transient radical species these leaving electrons produce, the so-called holes. Using pulse radiolysis measurements and first-principles calculations in the bulk and gas phases to interpret these, we show that the most likely hole species in this IL is a dimer radical anion that, somewhat surprisingly, has similar spectral features to the excess electron, including a broad near-infrared absorption band.

## Graphical abstract

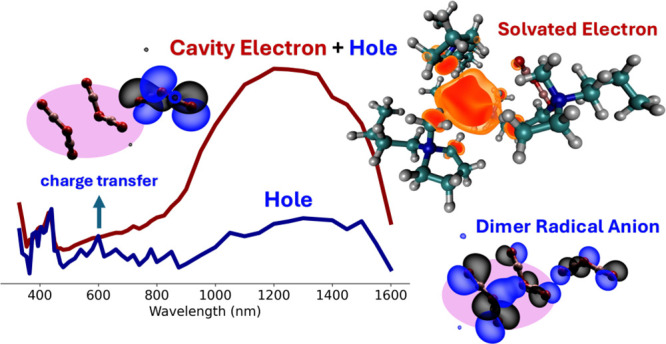

## 1 Introduction

In a conventional liquid, when a molecule loses an electron to radiation or photolysis, what is left behind is nominally a transient radical cationic species. Instead, in an ionic liquid (IL) or in molten salts (MSs), we commonly detach electrons from anions, leaving behind what can formally be described as a neutral radical, but in practice may be other species such as dimer radical anions^1–10^ that can then undergo further reactions. Figure 1 shows an example of this in two different ways for 1-butyl-1-methylpyrrolidinium dicyanamide ([Pyrr_1,4_][N(CN)_2_]), a popular IL due to its low viscosity and large electrochemical window.

**Figure 1:**
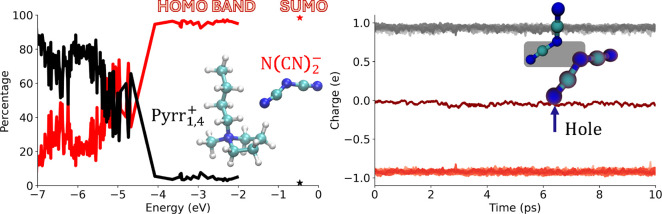
(Left) Typical projected density of states (PDOS) for the [Pyrr_1,4_]­[N­(CN)_2_] liquid in which an electron has been removed, creating a hole species. The PDOS was computed at the PBE0 level of theory using 40% Hartree–Fock exchange (HFX), as coded in the software CP2K^13^ (see Section 2). The black and red lines correspond to percentage projection onto Pyrr_1,4_^+^ and N­(CN)_2_^–^ components, respectively (ions shown as the inset). (Right) Löwdin charges of individual species computed along the AIMD trajectory using the DC-r^2^SCAN method, as coded in CP2K^13^ (see Section 2); gray-toned lines are for cations, red-toned lines for anions, and the single darker red line close to zero charge is for N­(CN)_2_^·^. The inset shows the dimer radical anion (N­(CN)_2_)_2_^·–^, highlighting that only one of the two components (N­(CN)_2_^·^) carries zero charge.

The right panel in Figure 1 shows the charge of condensed-phase species as a function of time from ab initio molecular dynamics (AIMD) simulations (see Section 2 for full methodological details), all of them approaching ±1 values except for a single charge-depleted radical species with zero charge. This indicates that the species that lost an electron to nominally form N­(CN)_2_^·^ was a N­(CN)_2_^–^ anion. The density of states projected onto Pyrr_1,4_^+^ (black) and N­(CN)_2_^–^ (red) species depicted on the left panel of Figure 1 also confirms that the singly unoccupied molecular orbital (SUMO), for which cationic and anionic contributions are denoted with a star, has, for all practical purposes, no contribution from Pyrr_1,4_^+^ species. In fact, the cationic contribution to the whole highest occupied molecular orbital (HOMO) band is almost negligible, once again showing that electrons detach at the lowest energy from anions in this system. The fact that the HOMO band is dominated by the anions is consistent with prior work using a simpler level of theory.^11,12^

When considering the spectroscopy of charge-depleted radical species in condensed-phase [Pyrr_1,4_]­[N­(CN)_2_], transitions below ∼4 eV are for the most part expected to be those of lower energy electrons into the SUMO and not transitions across the liquid band gap, which occur at higher energy.^12,14^ In other words, the spectroscopy that we are interested in probing is that which moves the hole downward in energy; this is why on the left panel of Figure 1, we only show the SUMO orbital energy and that of orbitals that are lower in energy compared to it.

Section 2 describes experimental and computational methodology used in the study, and Section 3 describes our interpretation of the transient spectroscopic features of hole species in [Pyrr_1,4_]­[N­(CN)_2_].

## 2 Methods

Section 2.1 provides details about sample handling and pulse radiolysis, and Section 2.2 describes our computational methodology.

### 2.1 Pulse Radiolysis

Neat [Pyrr_1,4_]­[N­(CN)_2_] was purchased from Iolitec and used in our pulse radiolysis experiments following the same purification procedure using activated charcoal, as described in ref 15. Purified samples were dried overnight in a vacuum oven at 45 °C, purged with Ar for 20 min, and transferred to an Ar-filled glovebox. To prepare samples for electron-scavenging experiments, dichloromethane (DCM, Acros Organics, 99.9%, extra dry, stabilized, used as received) and [Pyrr_1,4_]­[N­(CN)_2_] were added to a 1 mL volumetric flask to make a 500 mM DCM concentration. Lower concentrations were prepared by combining volumes of 500 mM DCM and neat [Pyrr_1,4_]­[N­(CN)_2_] in separate vials. Masses of each volume added were measured on an analytical balance. Samples of each concentration were then placed in septum-capped 5 mm path length Suprasil spectrophotometer cuvettes and kept in the glovebox prior to experiments.

The Laser Electron Accelerator Facility (LEAF) at Brookhaven National Laboratory was used to perform pulse radiolysis experiments.^16^ Electron pulse durations were less than 50 ps and delivered an average of 3.6 Gy/pulse. Radiation dosimetry was carried out at ambient temperature using the absorbance of the (SCN)_2_^·–^ radical at 470 nm in a standard aqueous solution of 10 mM KSCN saturated with N_2_O (Gϵ = 5.08 × 10^4^). Analyzing light from 310 to 1600 nm was provided by a pulsed xenon arc lamp. Specific wavelengths were selected using bandpass interference filters (10 nm below 800 and 25 nm at 800 nm and above, Edmund Optics). The selected wavelengths were detected by either silicon (300–1000 nm, FND-100Q, Excelitas Technologies) or germanium (900–1600 nm, GEP-600, GPD Optoelectronics Corp) photodiodes. Signals were digitized using a Teledyne LeCroy WaveRunner 640Zi 4 GHz, 8-bit, HDO6104 1 GHz, 12-bit oscilloscope. Data were processed using customized LabVIEW (National Instruments) and Igor Pro (Wavemetrics, Inc.) software routines.

### 2.2 Computational Methodology

Below we describe the classical pre-equilibration protocol and first-principles studies as well as the calculation of optical spectra.

#### 2.1.1 Classical Molecular Dynamics

The classical pre-equilibration protocol for the current study closely follows that described in our prior publication^15^ for an excess electron in [Pyrr_1,4_]­[N­(CN)_2_]. Force field parameters are the same as in ref 15. We used the GROMACS-5.1.4 simulation package;^17–19^ the simulation box comprised 16 ion pairs (a total of 560 atoms) with an initial structure prepared using the Packmol software.^20^ The system went through an initial energy minimization and a sequence of molecular dynamics (MD) steps to finally reach equilibrium at 300 K and 1 bar. The protocol involved a gradual scaling of charges: starting with 1% for 2 ns at 50 bar, then increasing to 10% for another 2 ns at 50 bar, and finally reaching 100% for 2 ns at 1 bar under constant temperature and pressure (NPT) conditions. The system was subsequently subjected to simulated annealing for 10 ns at 1 bar under the same NPT ensemble, with the temperature gradually increasing from 300 to 600 K and then decreasing to 300 K at full charge. After the annealing step, a final 10 ns simulation was performed at 300 K under identical NPT conditions. During the initial equilibration steps, the V-rescale thermostat^21^ (with a 0.2 ps time constant) and the Berendsen barostat^22^ (with a 1.0 ps time constant) were employed. For the annealing and final production run, the Nosé–Hoover thermostat^23^ (0.2 ps time constant) and the Parrinello–Rahman barostat^24^ (1.0 ps time constant) were used. All simulations utilized the MD integrator implemented in GROMACS. A cutoff distance of 0.75 nm was applied to all nonbonded interactions. Electrostatic interactions were calculated using the Particle-Mesh Ewald method,^25,26^ with a Fourier grid spacing of 0.08 nm and sixth-order interpolation.

#### 2.1.2 First-Principles Studies and the Calculation of the Optical Spectra

A classical simulation frame was taken from the final run that closely matched the average equilibrated density. This frame was used as input for a first-principles conjugate gradient energy optimization (PBE-D3 level of theory,^27–30^ spin-restricted, 600 Ry planewave cutoff, DZVP basis sets and GTH pseudopotentials, as implemented in CP2K^31,32^ ). The intention was not to fully optimize the condensed phase system to the bottom of the local potential energy surface but instead to bring bond lengths and angles from their preferred classical values to those preferred by the first-principles potential. Using the same level of theory, we then carried out a 20 ps constant temperature and volume AIMD equilibration in which the box had zero charge and the multiplicity of the system was one.

Using the final frame in the previous step, a positive charge was introduced (at this point, the system had a multiplicity of two and was treated as spin unrestricted), and a final 10 ps AIMD trajectory was run using the density-corrected r^2^SCAN (DC-r^2^SCAN) method.^33,34^ The choice to use DC-r^2^SCAN was made to eliminate possible density-driven delocalization error;^35,36^ that we have seen in prior work,^11^ where simulations including neutral radicals can result in significant hole delocalization. Such delocalization could also be mitigated, albeit at a higher computational cost, by increasing the share of Hartree–Fock exchange (HFX) component of hybrid functionals during AIMD. We had excellent results using DC-r^2^SCAN in our recent work on an excess electron in the same system^15^ and follow the same strategy here for consistency.

All DC-r^2^SCAN calculations were performed with a 1200 Ry planewave cutoff, a 6 Å cutoff radius for the truncated Coulomb potential, and a Schwarz integral threshold of 1 × 10^–7^. We employed the TZV2P basis set in conjunction with GTH-SCAN pseudopotentials, along with the cpFIT3 ADMM basis set for constructing auxiliary density matrices.^37^

For the final 0.6 ps of the DC-r^2^SCAN trajectory, condensed-phase spectra were computed every 0.1 ps, with 50 excitations calculated per spectrum. (Each of these seven calculations, which require very large memory nodes, takes approximately 2 weeks on two nodes each with 128 threads and 1.5 TB of memory). The calculations were carried out at the PBE0-D3 level with 40% HFX, using an 800 Ry planewave cutoff, TZVP basis sets combined with GTH-PBE0 pseudopotentials, and admm-tzp basis sets to reduce computational cost. All TDDFT calculations were performed using the Tamm–Dancoff approximation. In addition to the 50 excitations calculations and for better statistics, 21 TDDFT calculations comprising 20 excitations only associated with the last 2 ps of simulation were also performed using the same level of theory. Figure S1 shows the outcome of these calculations, in which only regions A and B of the spectrum, but not region C (vide infra), are fully covered.

The choice of 40% HFX for spectral calculations was made based on the fact that a higher percentage of HFX results in undesirably blue-shifted spectral features for the excess hole when compared to experiments. For this specific system, PBE0-D3 with 40% HFX generates fewer blue-shifted spectral features while still maintaining minimal spin leakage of the radical dianion.

In addition to condensed-phase spectra, gas-phase TDDFT calculations at the WB97XD/def2-TZVP level of theory using the Gaussian09W package^38^ were performed on structures of (N­(CN)_2_)_3_^·2–^ derived from the condensed-phase trajectory. These TDDFT calculations are significantly less computationally expensive, and we took advantage of this to obtain good statistics in the gas-phase spectrum presented in the bottom left panel of Figure 3. An extra advantage of these gas-phase calculations is that, by including only molecules of interest, the spectral analysis was significantly simplified.

## 3 Results and Discussion

Before discussing our spectroscopic results and their interpretation using first-principles calculations in the condensed phase, it is instructive to understand the nature of (N­(CN)_2_)_2_^·–^, the species^1,8^ that more accurately represents the hole in this system. Figure 2 shows the gas-phase spectra of three minimized (N­(CN)_2_)_2_^·–^ conformers, each characterized by the type of alignment of N atoms in what nominally can be described as an association of a N­(CN)_2_^–^ anion and a N­(CN)_2_^·^ radical. For each case, the figure also shows orbitals that are involved in low-energy transitions such as $\mathrm{SUMO}‐1\overset{\left[h\nu \right]}{\to }\mathrm{SUMO}$ . Note how these orbitals link N atoms, and to a lesser extent C atoms, across molecules. When inspected carefully, one can think of (N­(CN)_2_)_2_^·–^ as having a hemibond^39^ or similar labile three-electron bond. The consequence of this in the condensed phase is that the N­(CN)_2_^–^ component of (N­(CN)_2_)_2_^·–^ can easily exchange on a picosecond time scale.

**Figure 2:**
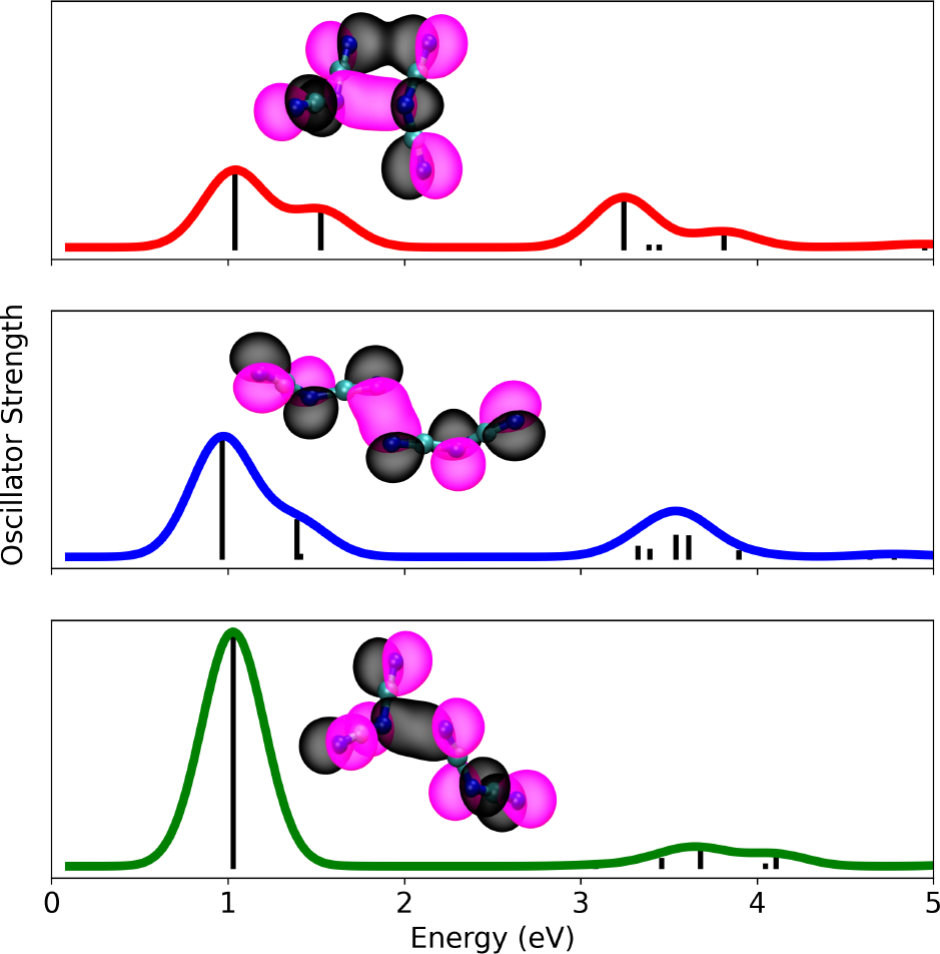
Gas-phase optimized conformations (B3LYP-D3/def2svp) of the (N­(CN)_2_)_2_^·–^ dimer radical anion differing in the alignment of N atoms across N­(CN)_2_^–^ and N­(CN)_2_^·^ species, and their corresponding line spectrum broadened for visual impact. Molecular orbitals shown as insets correspond to the SUMO-1, which is involved in the lowest energy transition $\mathrm{SUMO}‐1\overset{\left[h\nu \right]}{\to }\mathrm{SUMO}$ . Molecular orbitals were computed using the WB97XD/def2tzvp method; spectra are from TDDFT calculations using the same method. Calculations were carried out using the Gaussian Software.^38^

The upper left panel in Figure 3 shows pulse radiolysis spectra (measured at the BNL LEAF Facility^16^) of [Pyrr_1,4_][N(CN)_2_] at 10 ns across a range of concentrations (0–500 mM) of the electron-scavenger DCM. The spectrum labeled neat is that of pure [Pyrr_1,4_]­[N­(CN)_2_] (0 mM DCM) and includes both the spectral features of the excess electron and the excess hole. As the concentration of the DCM scavenger increases, electron-scavenging efficiency increases, and the spectral contributions associated with the excess electron decrease. Between the two highest DCM concentrations, 250 and 500 mM, there is no additional change in the spectrum, and it is reasonable to assume that the vast majority of initially formed excess electrons have been removed by the scavenger within 10 ns. The excess electron displays a prominent near-infrared (NIR) band in the spectral region labeled **A**, and we have thoroughly discussed its origin in ref 15. Interestingly, the experimental spectrum of the electron-quenched IL still shows a broad NIR band in region **A**, albeit of significantly lower intensity; we argue that the feature includes contributions from (N­(CN)_2_)_2_^·–^. A major fraction of the intensity in regions **B** and **C** of the experimental spectrum of neat [Pyrr_1,4_]­[N­(CN)_2_] after irradiation remains upon quenching of the electrons, implying that these have important contributions from species that are not excess electrons. As will become clear, these are also prominently detected computationally when we study the radical dimer anion (N­(CN)_2_)_2_^·–^.

**Figure 3:**
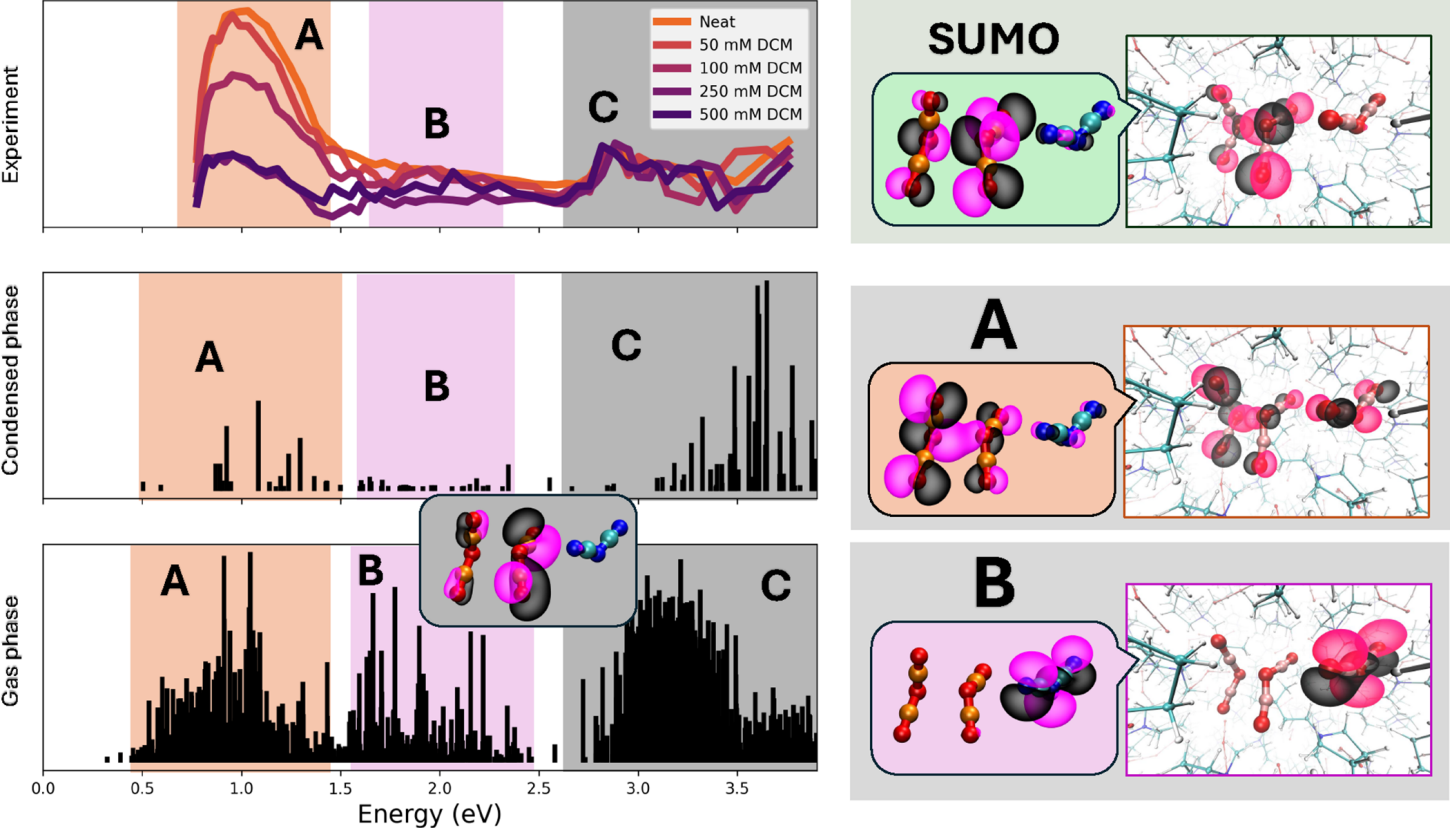
(Top left) Experimental optical spectra for different concentrations of the electron-scavenger DCM in [Pyrr_1,4_]­[N­(CN)_2_] at 10 ns after irradiation. Highlighted regions **A**, **B**, and **C** indicate distinct spectroscopic transition regions. (Center left) A combined set of seven-line spectra were computed from our condensed-phase simulations every 0.1 ps, between 9.4 and 10 ps, each with 50 excitations. Spectra were computed using CP2K^13^ at the PBE0-D3 40% HFX level of theory (see Section 2). (Bottom left) Combined gas-phase spectra for (N­(CN)_2_)_2_^·–^ and an extra N­(CN)_2_^–^ anion were obtained using the WB97XD method as coded in the Gaussian software.^38^ The gas-phase configurations were taken from the condensed phase run (181 simulation frames between 1 and 10 ps); each calculation included 20 excitations. (Top right) SUMO computed in the gas and condensed phases. (Center right) Typical orbital computed in the gas and condensed phases, from which excitation into the SUMO results in transitions in region **A**. (Bottom right) Typical orbital computed in the gas and condensed phases from which excitation into the SUMO results in transitions in region **B**. Inset (bottom left) a typical originating orbital involved in an intramolecular transition of N­(CN)_2_^·^ in region **C** computed in the gas phase.

The center left panel in Figure 3 shows the combined condensed-phase spectra from a series of simulation snapshots of liquid [Pyrr_1,4_]­[N­(CN)_2_] in which an electron had been initially removed to create a hole. Regions **A**, **B**, and **C** matching the experimental electron-quenched spectrum can be clearly discerned (see also Figure S1 for a spectrum that includes more simulation snapshots but less excitations per snapshot, providing better statistics in regions **A** and **B** but which do not fully cover region **C**).

The bottom left panel in Figure 3 shows the combined gas-phase spectrum of 181 frames, including (N­(CN)_2_)_2_^·–^ and an extra N­(CN)_2_^–^; the combined gas-phase spectrum reproduces all three spectral features **A** through **C**. The extra N­(CN)_2_^–^ is needed to recover band **B**; this is because not all transitions in the spectrum are of (N­(CN)_2_)_2_^·–^ alone. Some involve charge transfer from adjacent N­(CN)_2_^–^ in the solvent into (N­(CN)_2_)_2_^·–^. The result of such a solvent to radical charge transfer is an equivalent (N­(CN)_2_)_2_^·–^, as described in the equation

$$N(\mathrm{CN}{)}_{2}^{-}-N(\mathrm{CN}{)}_{2}^{\cdot }+N(\mathrm{CN}{)}_{2}^{-}\overset{\left[h\nu \right]}{\to }N(\mathrm{CN}{)}_{2}^{-}+N(\mathrm{CN}{)}_{2}^{-}-N(\mathrm{CN}{)}_{2}^{\cdot }$$

Alternatively, one could argue that transiently the hole species is (N­(CN)_2_)_3_^·2–^, and that transitions in band **B** move an electron from the particle state to the hole state within this larger (N­(CN)_2_)_3_^·2–^ aggregate. Computational evidence that a solvent N­(CN)_2_^–^ is required for band **B** to appear is shown in Figure 2, where all gas-phase (N­(CN)_2_)_2_^·–^ conformers display bands **A** and **C** but not **B**.

Center and bottom panels on the right in Figure 3 show typical originating electronic states for transitions in the **A** and **B** bands, respectively; the landing state is always the SUMO that is depicted on the top panel. A typical band **A** transition originates from a bonding or an antibonding orbital linking N­(CN)_2_^·^ and N­(CN)_2_^–^; some examples of these are depicted in the insets of Figure 2. Instead, a typical **B** band transition corresponds to electron transfer from an adjacent N­(CN)_2_^–^ ion into the SUMO of (N­(CN)_2_)_2_^·–^. High-intensity transitions in band **C** are often intramolecular of N­(CN)_2_^·^ (see a possible originating orbital depicted as an inset in the bottom left panel in Figure 3), but we do not discount the possibility that other types of transitions can also occur in this higher-energy regime.

As a final check into our results, we briefly looked at the spectroscopy of CH_2_Cl^·^, the expected product of the excess electron reaction with the scavenger (data not shown). Using the same methodology for our gas-phase calculations described earlier, we found that excitations of this species are at energies significantly larger than those studied here. When considering possible solvent to radical charge transfer transitions between N­(CN)_2_^–^ and CH_2_Cl^·^, we found that those appear to be above 3.7 eV but do not discard that they could potentially overlap with the highest energy portion of region **C** in Figure 3.

## 4 Conclusions

We find that the most likely energy-relaxed hole species left behind after electron detachment caused by irradiation in [Pyrr_1,4_]­[N­(CN)_2_] is a dimer radical anion. This dimer radical anion has a labile bond, and because of this, its N­(CN)_2_^–^ component exchanges upon solvent fluctuations on a picosecond time scale. Three spectroscopic bands are observed in the relevant 0–4 eV regime. Interestingly, one of these is in the NIR, overlapping with the characteristic absorption band of the cavity electrons in [Pyrr_1,4_]­[N­(CN)_2_]. This NIR band appears to be from the hemibond into the SUMO. Other bands can be ascribed as a solvent-to-radical charge transfer and intramolecular transitions of N­(CN)_2_^·^.

## Supplementary Material



## Notes

Additional TDDFT spectra computed with 20 excitations only (PDF)
