# Supplementary material for: Ionic Liquids under Radiation and the Dimer Radical Dicyanamide Anion
Source: J Phys Chem B. 2025 Nov 13;129(47):12261–6. doi: 10.1021/acs.jpcb.5c07381 (PMC12670383; doi:10.1021/acs.jpcb.5c07381)
Supplement: Supplementary file 1 [file jp5c07381_si_001.pdf]

Supporting Information: Ionic Liquids Under Radiation and the Dimer Radical Dicyanamide Anion

Hung H. Nguyen,[†,§] Katie Huber,[‡,§] Dishan Das,[†] James F. Wishart,[*,¶] David A. Blank,[*,‡] and Claudio J. Margulis[*,†]

†*Department of Chemistry, The University of Iowa, Iowa City, Iowa 52242, United States*

‡*Department of Chemistry, University of Minnesota, Minneapolis, MN 55455, United States*

¶*Chemistry Department, Brookhaven National Laboratory, Upton, NY 11973, United States*

§*These authors contributed equally.*

E-mail: wishart@bnl.gov; blank@umn.edu; claudio-margulis@uiowa.edu

This Supporting Information provides a supporting figure.

S.1 Supporting Figure

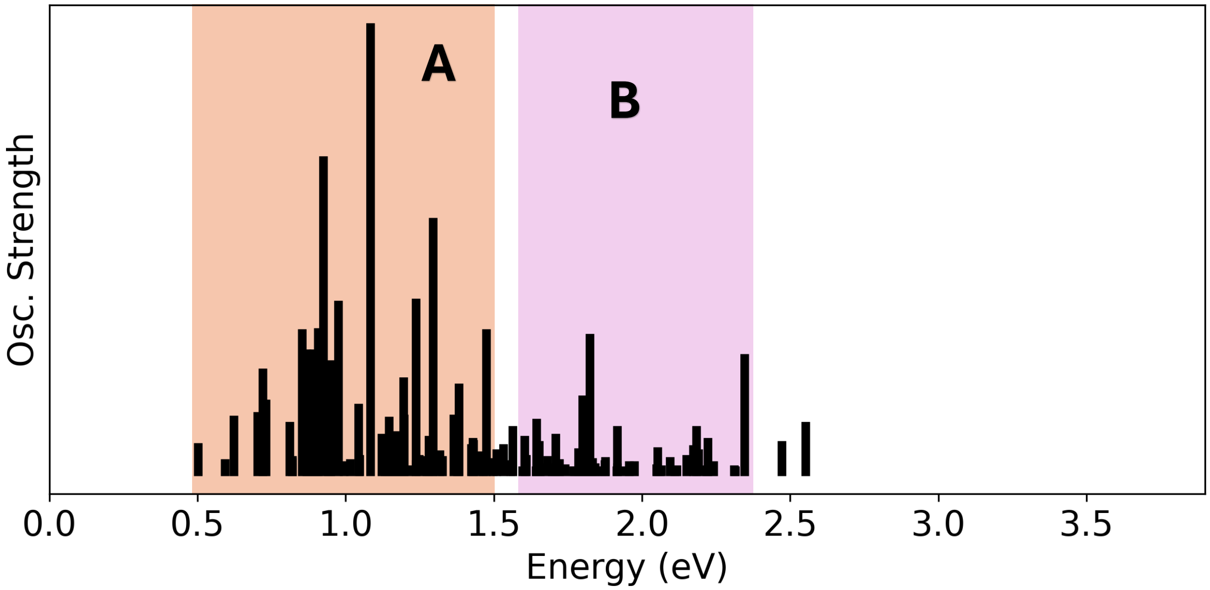

Figure S1: Combined set of 21 line spectra from our condensed-phase simulations every 0.1 ps, between 8-10 ps, each with 20 excitations. TDDFT spectrum at the PBE0-D3 40% HFX level of theory, computed in the condensed-phase using CP2K.[S1] This spectrum should be contrasted with that in the middle panel of Figure 3 in which 50 excitations are included.

References

(S1) Kühne, T. D.; Iannuzzi, M.; Del Ben, M.; Rybkin, V. V.; Seewald, P.; Stein, F.; Laino, T.; Khaliullin, R. Z.; Schütt, O.; Schiffmann, F.; Golze, D.; Wilhelm, J.; Chulkov, S.; Bani-Hashemian, M. H.; Weber, V.; Borštnik, U.; Taillefumier, M.; Jakobovits, A. S.; Lazzaro, A.; Pabst, H.; Müller, T.; Schade, R.; Guidon, M.; Andermatt, S.; Holmberg, N.; Schenter, G. K.; Hehn, A.; Bussy, A.; Belleflamme, F.; Tabacchi, G.; Glöß, A.; Lass, M.; Bethune, I.; Mundy, C. J.; Plessl, C.; Watkins, M.; VandeVondele, J.; Krack, M.; Hutter, J. CP2K: An

electronic structure and molecular dynamics software package - Quickstep: Efficient and accurate electronic structure calculations. *J. Chem. Phys.* **2020**, *152*, 194103.
